# Pregnancy Is Enough to Provoke Deleterious Effects in Descendants of Fructose-Fed Mothers and Their Fetuses

**DOI:** 10.3390/nu13103667

**Published:** 2021-10-19

**Authors:** Elena Fauste, María I. Panadero, Cristina Donis, Paola Otero, Carlos Bocos

**Affiliations:** Facultad de Farmacia, Universidad San Pablo-CEU, CEU Universities, Montepríncipe, Boadilla del Monte, 28668 Madrid, Spain; ele.fauste.ce@ceindo.ceu.es (E.F.); ipanade@ceu.es (M.I.P.); cristinadonis9@gmail.com (C.D.); paotero@ceu.es (P.O.)

**Keywords:** fructose, pregnancy, fetal programming, fetus, lipids, insulin, leptin

## Abstract

The role of fructose in the global obesity and metabolic syndrome epidemic is widely recognized. However, its consumption is allowed during pregnancy. We have previously demonstrated that maternal fructose intake in rats induces detrimental effects in fetuses. However, these effects only appeared in adult descendants after a re-exposure to fructose. Pregnancy is a physiological state that leads to profound changes in metabolism and hormone response. Therefore, we wanted to establish if pregnancy in the progeny of fructose-fed mothers was also able to provoke an unhealthy situation. Pregnant rats from fructose-fed mothers (10% *w/v*) subjected (FF) or not (FC) to a fructose supplementation were studied and compared to pregnant control rats (CC). An OGTT was performed on the 20th day of gestation, and they were sacrificed on the 21st day. Plasma and tissues from mothers and fetuses were analyzed. Although FF mothers showed higher AUC insulin values after OGTT in comparison to FC and CC rats, ISI was lower and leptinemia was higher in FC and FF rats than in the CC group. Accordingly, lipid accretion was observed both in liver and placenta in the FC and FF groups. Interestingly, fetuses from FC and FF mothers also showed the same profile observed in their mothers on lipid accumulation, leptinemia, and ISI. Moreover, hepatic lipid peroxidation was even more augmented in fetuses from FC dams than those of FF mothers. Maternal fructose intake produces in female progeny changes that alter their own pregnancy, leading to deleterious effects in their fetuses.

## 1. Introduction

During pregnancy, maternal health is essential for the correct development of the progeny. In fact, many studies have demonstrated how an inappropriate nutrition and/or diseases such as obesity and diabetes during pregnancy can promote metabolic and cardiovascular disturbances in the offspring when adults [1]. The mechanism underlying this effect is called fetal programming [2]. The placenta plays a key role in this process, since it regulates the correct supply of nutrients and protects the fetus ensuring correct fetal development [3]. Both mother malnutrition and overnutrition during pregnancy can cause deleterious effects in the progeny such as defective hormonal responses, oxidative stress and epigenetic modifications [4,5]. Moreover, a higher risk of cardiovascular diseases and diabetes in descendants from obese or diabetic mothers has been well-described in the bibliography [6,7].

The predictive adaptive response hypothesis states that animals whose mothers have been fed a high-sucrose diet (HSD) would exhibit a more favorable metabolic profile in the presence of an HSD in their adult life. However, several reports showed that maternal high sugar feeding did not protect the progeny from carbohydrate-induced metabolic disturbances [8,9,10]. Furthermore, fetal programming remains active generation after generation when the exposure to the stress factor occurs during critical periods of life, such as pregnancy and lactation [11]. During these periods, when pregnant subjects (F0) are in contact with the stress factor, their female descendants (F1) (as fetuses) are also in contact with the factor. Thus, when this “first generation” (F1) becomes pregnant, both, if they are again in contact with the stress factor or not, the metabolic health of their descendants (F2) is already affected, and so on, successively [12,13]. However, long-term rodent studies following the offspring into adulthood and pregnancy are still lacking.

Fructose such as High-Fructose Corn Syrup (HFCS) has been extensively used as added sugar in sugar-sweetened beverages (SSB) and processed foods due to its higher sweetening power and its higher solubility in water. HFCS has even replaced sucrose as the main added sugar in the USA [14]. Interestingly, a relationship between fructose intake and higher rates of obesity [15], metabolic syndrome (MetS) [16], non-alcoholic fatty liver disease (NAFLD) [17], and insulin resistance [18] has been well established. Moreover, we and others have demonstrated in animal model studies that fructose intake during pregnancy can cause metabolic disturbances in the offspring, even when they become adults [19,20,21,22]. However, the ingestion of SSB and foods rich in fructose is still allowed during gestation.

The effects of fructose intake during gestation have previously been studied using sucrose-rich diets. Thus, a high amount of sucrose (versus an equal amount of cornstarch) produced maternal hypertriglyceridemia that may have contributed to the negative effects of sucrose on the developing fetus [23]. Rodent studies have demonstrated that fructose in pregnancy can also significantly reduce the weight of the placenta [24]. Interestingly, the influence of maternal sugar consumption on obesity, insulin resistance, and cardiovascular disease in offspring and mothers has been previously reviewed. Thus, several animal studies found that maternal diets consisting of 75% dextrose and maltodextrin led to a higher body weight and an insulin resistant state in both mothers and offspring when compared to mothers consuming a diet composed of 35% simple carbohydrates. In fact, these authors have proposed that maternal diets throughout pregnancy which are high in fructose have damaging effects on maternal and offspring health similar to the high dextrose/maltodextrin enriched diets [25]. However, other authors, in a report comparing dietary glucose versus fructose, concluded that, during pregnancy, it was the quantity rather than quality of carbohydrate that determined fetal and postnatal development [26].

Previously, we found that maternal fructose intake provoked hypertriglyceridemia, liver triglyceride accumulation, and a diminished leptin response in mothers, along with an impaired leptin signaling and hepatic steatosis in their fetuses [23]. Subsequently, fructose in pregnancy led to impaired insulin signaling and hypoadiponectinemia in adult male progeny. Interestingly, adult females from fructose-fed mothers did not exhibit any of these disturbances [27]. However, we thought that, in reality, these female rats kept a programmed phenotype hidden. In fact, when the female progeny born of fructose-fed mothers were supplemented with fructose when adults, clear dyslipidemia and liver steatosis were observed in comparison to descendants from control mothers or progeny not subjected to fructose feeding [28].

Therefore, in the present work, we wanted to answer two questions: (1) given that pregnancy is a physiological state that produces profound changes in lipids and glucose metabolism and insulin and leptin responses in dams, would pregnancy alone be sufficiently able to reveal the programmed phenotype hidden in females from fructose-fed dams? and (2) since we observed in non-pregnant rats that the programmed phenotype was initially hidden but appeared when fructose was offered to descendants of fructose-fed mothers [28], what effects would this re-exposure to fructose produce in females from fructose-fed dams when they were pregnant? Finally, we evaluated whether this situation could also affect their fetuses and we studied how the placenta was involved in this process.

## 2. Materials and Methods

### 2.1. Animals and Experimental Design

Female Sprague-Dawley rats weighing 200–240 g were fed ad libitum a standard rat chow diet (Teklad Global 14% Protein Rodent Maintenance Diet, Envigo, Indianapolis, Indiana), and housed under controlled light and temperature conditions (12-h light-dark cycle; 22 ± 1 °C). The experimental protocol was approved by the Animal Research Committee of the University San Pablo-CEU, Madrid, Spain (ref. numbers 10/206458.9/13 and 10/042445.9/19). Animals were mated, and day 0 of pregnancy was determined by the appearance of spermatozoids in vaginal smears. The experimental protocol to which pregnant rats (F0 generation) were subjected was the same as previously reported [23]. Briefly, pregnant rats were randomly separated into a control group (no supplementary sugar in drinking water) and a fructose-supplemented group (fructose 10% wt/vol in drinking water) throughout gestation (five rats per group). Pregnant rats were allowed to deliver, and, on the day of birth, each suckling litter was reduced to nine pups per mother. After delivery, both mothers and their pups were maintained with water and food ad libitum. At 21 days of age, pups were separated by gender and female progeny continued to be fed on a standard rat chow diet and tap water with no additives.

When female progeny (F1 generation) from control and fructose-fed mothers reached 8 weeks of age, they were mated and day 0 of gestation was determined by the appearance of spermatozoids in vaginal smears. Then, pregnant rats from control mothers were kept on solid pellets and supplied with tap water with no supplementary sugar during gestation and considered the CC group. On the other hand, pregnant rats from fructose-fed mothers were randomly separated into two groups. In order to minimize the “litter effect”, animals within each experimental group were born to different dams. Thus, one set of pregnant rats were fed a standard rat chow diet and received water with no additives (FC group) and another half of pregnant rats drank fructose at 10% wt/vol in drinking water (FF group) throughout gestation (five rats per group). Thus, three experimental groups were established: the first letter indicating whether the mothers (F0 generation) had been supplied with tap water during pregnancy (C, control) or water containing fructose (F, fructose); and the second letter indicating whether the progeny (F1 generation) received fructose (F) or not (C) during their own pregnancy. Intake of solid food and liquid per cage were recorded daily and the area under the curve (AUC) for the food and liquid ingested and total calories were calculated.

Pregnant rats were decapitated on the 21st day of gestation at 10:00 o’clock. Prior to sacrifice, food was removed at 8:00 o’clock. Blood was collected into EDTA-containing tubes, plasma was obtained by centrifugation and stored at −20 °C until processed. The conceptus was dissected, and after being weighed, fetuses were counted and weighed. Placentas and livers were also obtained, weighed and frozen. Fetuses (F2 generation) were decapitated, and blood from all pups of the same mother was collected and pooled into tubes containing EDTA to obtain plasma. The livers of the fetuses were extracted, and those coming from the same mother were pooled and placed in liquid nitrogen to be stored at −80 °C until processed for further analysis.

A second set of rats was subjected to the same protocol as mentioned above and on the morning of the 20th day of pregnancy, five rats per group (CC, FC and FF) were subjected to an oral glucose tolerance test (OGTT) in fasted conditions (12-h fasting). A basal blood sample from the tail vein was taken and a bolus of glucose (2 g/kg) was orally administered to the animals. Subsequently, blood samples were collected into EDTA tubes at 7.5, 15, 22.5, 30, and 60 min after glucose administration. Samples were then centrifuged, and plasma was stored at −20 °C until processed, determinations were made and the AUC values for glucose and insulin were calculated.

### 2.2. Plasma Determinations

Plasma aliquots were used to determine glucose, cholesterol and triglycerides (Spinreact, Girona, Spain), and non-esterified fatty acids (NEFA) (Wako, Japan) using commercial kits. Specific ELISA kits for rats were used to measure insulin (Mercodia, Uppsala, Sweden), leptin (Biovendor, Brno, Czech Republic), and adiponectin (Merck-Millipore, Bedford, MA, USA) following the manufacturer instructions. Insulin Sensitivity Index (ISI) was calculated as the ratio 2/[(plasma insulin μM × plasma glucose μM) + 1] [29].

### 2.3. Liver and Placenta Determinations

Two hundred milligrams of frozen liver or placenta were immersed in chloroform:methanol 2:1 plus dibutylhydroxytoluene (BHT), and used for lipid extraction following the Folch method [30]. Aliquots of lipid extracts were dried, and the remaining residue was weighed to determine total lipid content. Four milliliters of lipid extracts were dried and redissolved in isopropanol to determine cholesterol using an enzymatic colorimetric assay (Spinreact, Girona, Spain). Triglycerides were also measured using the procedure described by Carr et al. [31]. Briefly, 1 mL of Triton-X 100 1.25% in chloroform was added to 0.6 mL of lipid extracts, dried and resuspended in 0.5 mL of distilled water. Triglycerides were measured using an enzymatic colorimetric assay as mentioned above.

One hundred milligrams of liver or placenta were homogenized in 1.2 mL phosphate buffered saline (PBS). After centrifugation, 10 μL of dibutylhydroxytoluene (BHT) were added to avoid lipid oxidation. These homogenates were used to measure malondialdehyde (MDA) as a marker of lipid peroxidation. The method was previously described by Wong et al. [32] and MDA-thiobarbituric acid complexes were measured by fluorescence at 515 nm/553 nm excitation/emission wavelengths. These homogenates were also used after deproteination with trichloroacetic acid (TCA) 20% to measure glucose using specific enzymatic colorimetric assays (Spinreact, Girona, Spain).

### 2.4. RNA Extraction and Gene Expression Determination by qPCR

Total RNA was isolated from liver or placenta using Ribopure (Invitrogen, ThermoFisher Scientific, Waltham, MA, USA). Total RNA was subjected to DNase I treatment using Turbo DNA-free (Invitrogen, ThermoFisher Scientific, USA), and RNA integrity was confirmed by agarose gel electrophoresis. Afterwards, cDNA was synthesized by oligo(dT)-primed reverse transcription with Superscript II (Invitrogen, ThermoFisher Scientific, USA). qPCRs were performed using a Light Cycler 1.5 (Roche, Mannheim, Germany). The reaction solution was carried out in a volume of 20 μL, containing 10 pmol of both forward and reverse primers, 10× SYBR Premix Ex Taq (Takara Bio Inc., Shiga, Japan) and the appropriate nanograms of the cDNA stock. Rps29 was used as a reference gene for qPCR. The primer sequences were obtained either from the Atlas RT-PCR Primer Sequences (Clontech, Palo Alto, CA, USA) or designed using the Primer3 software (University of Massachusetts Medical School, Worcester, MA, USA) [33]. Samples were analyzed in duplicate on each assay. Amplification of non-specific targets was discarded using the melting curve analysis method for each amplicon. qPCR efficiency and linearity were assessed by optimization of the standard curves for each target. The transcription was quantified by the Light Cycler Software 4.05 (Roche, Germany) using the efficiency correction method [34].

### 2.5. Statistical Analysis

Results were expressed as means ± S.E. Treatment effects were analyzed by one-way analysis of variance (ANOVA). When treatment effects were significantly different (*p* < 0.05), means were tested by Tukey’s multiple range test, using the computer program SPSS (version 25). When the variance was not homogeneous, a post hoc Tamhane test was performed.

## 3. Results

### 3.1. Pregnancy in Progeny from Fructose-Fed Mothers with or without Fructose Intake throughout Gestation Alters Insulin Sensitivity, Leptin Response, and Liver and Placenta Lipid Contents

As previously reported [23,24,28], fructose intake produces a significant increase in the ingestion of liquids and a non-significant reduction in solid food consumption (Table 1), leading to a significantly higher total amount of ingested energy in fructose-fed dams (FF) versus the other two groups (Table 1). Curiously, in a previous report using descendants from control mothers, maternal fructose produced the same effect as that observed here, but the total amount of ingested energy was not significantly different versus controls (CF vs. CC) [23]. Remarkably, although the body weight (BW) at the beginning of the experiment was different between descendants from fructose-fed mothers and those from control mothers (FC and FF vs. CC), no changes in maternal BW increase (without conceptus) throughout gestation were found between the three groups evaluated (Table 1). Interestingly, despite the increased amount of calories ingested by the FF mothers, no changes in the conceptus weight, the number or the BW of their fetuses were observed when compared to CC and FC dams (Table 1).

**Figure 1:**
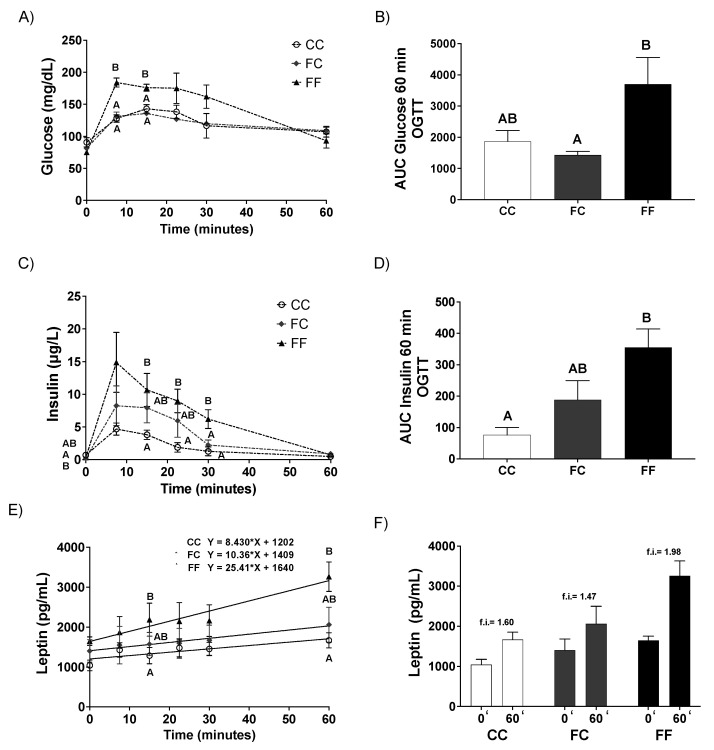
Ingestion of a 10% wt/vol fructose solution throughout gestation in pregnant rats from fructose-fed mothers deteriorates insulin and leptin responses of late pregnant rats. On the morning of the 20th day of pregnancy, animals were subjected to an OGTT in fasted conditions (12 h fasting). After a basal blood sample from the tail vein was drawn, a bolus of glucose (2 g/kg body weight) was administered orally to the rats. Subsequently, blood samples were collected at 7.5, 15, 20, 30, 45, and 60 min after glucose administration, and after the plasma analysis of glucose and insulin was performed, the area under the curve (AUC) for both parameters was calculated. (**A**) Plasma glucose during the OGGT of 20-day-pregnant rats from control mothers (CC, open circles), or pregnant rats from fructose-fed mothers subjected (FF, triangles) or not (FC, grey circles) to fructose intake throughout pregnancy. (**B**) AUC for glucose of CC (empty bar), FC (grey bar) and FF (black bar) 20-day-pregnant rats during the OGGT. (**C**) Plasma insulin during the OGGT of 20-day-pregnant rats from control mothers (CC, open circles), or pregnant rats from fructose-fed mothers subjected (FF, triangles) or not (FC, grey circles) to fructose intake throughout pregnancy. (**D**) AUC for insulin of CC (empty bar), FC (grey bar), and FF (black bar) 20-d pregnant rats during the OGGT. (**E**) Plasma leptin values at different times after the oral administration of a bolus of glucose solution (2 g/kg body weight) to 20-day-pregnant rats from control mothers (CC, open circles), pregnant rats from fructose-fed mothers subjected (FF, triangles) or not (FC, grey circles) to fructose intake throughout pregnancy. Solid lines and equations correspond to linear regressions made with the corresponding values of each experimental group. (**F**) Plasma leptin levels before (t0′) and 60 min (t60′) after receiving a bolus of glucose from CC (empty bar), FC (grey bar), and FF (black bar) pregnant rats. Data are mean ± S.E. from 5 animals per group. Values not sharing a common letter are significantly different (*p* < 0.05) between the three groups.

To investigate the insulin–glucose relationship, a glucose tolerance test (OGTT) at day 20 of gestation was performed. As shown in Figure 1A, plasma glucose levels were similar for all the time points of the experiment between CC and FC groups. However, FF rats showed a trend to an increase in plasma glucose levels when compared to the other two groups of mothers, turning out to be significantly higher at 7.5 and 15 min of the OGTT (Figure 1A). Thus, FF dams showed an increase in the AUC of plasma glucose concentration that became significant in comparison to FC dams (Figure 1B). According to the results observed for glycemia in FF rats, the curve for plasma insulin levels remained, as expected, higher in this experimental group during all the time points of the experiment versus the other two groups (Figure 1C). Surprisingly, FC pregnant rats also showed a non-significant increase compared to CC dams (Figure 1C). In accordance with this, the AUC of insulin levels (Figure 1D) confirmed the profiles found at the different time points of the OGTT.

We had discovered an unexpected finding in a previous report, where we demonstrated that fructose intake during gestation provoked a diminished maternal leptin response to fasting and refeeding [23]. In fact, in agreement with this, other reports found that fructose causes hyperleptinemia [35] and leptin resistance [36]. Therefore, we also measured leptin levels in the animals used for the glucose tolerance test in order to investigate whether these rats could also present any disturbance in leptin signaling. As shown in Figure 1E,F, leptin levels after an overnight fasting period did not change between the three groups at the beginning of the experiment (t = 0 min). However, at different time points after the intake of a bolus of glucose (2 g/kg), whereas leptinemia increased in CC and FC dams almost in parallel (slope = 8.43 and slope = 10.36, respectively), in FF pregnant rats leptinemia increased more than twice in comparison to the other two groups (slope = 25.41, that is, an average increase in leptin of 25.41 pg/mL per minute) (Figure 1E). Consequently, at 60 min after the bolus of glucose, leptinemia had similarly augmented in CC and FC rats (showing the same fold induction as previously observed for CC, [23]), while it had duplicated in FF rats (Figure 1F).

**Figure 2:**
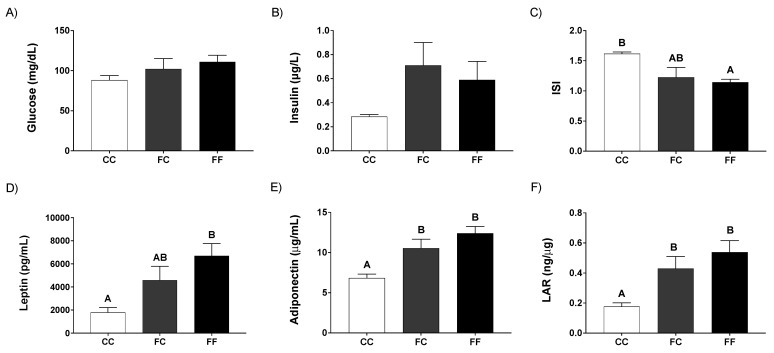
Pregnancy in progeny from fructose-fed mothers and ingestion of a 10% wt/vol fructose solution throughout gestation in pregnant rats from fructose-fed mothers affects maternal leptin and insulin responses. Plasma (**A**) glucose and (**B**) insulin, and (**C**) insulin sensitivity index (ISI) of 21-day-pregnant rats from control mothers (CC, empty bar), or pregnant rats from fructose-fed mothers subjected (FF, black bar) or not (FC, grey bar) to fructose intake throughout pregnancy. Plasma (**D**) leptin and (**E**) adiponectin, and (**F**) leptin/adiponectin ratio (LAR) of 21-day-pregnant rats from control mothers (CC, empty bar), or pregnant rats from fructose-fed mothers subjected (FF, black bar) or not (FC, grey bar) to fructose intake throughout pregnancy. Data are mean ± S.E. from 5 pregnant rats. Values not sharing a common letter are significantly different (*p* < 0.05) between the three groups.

Interestingly, plasma parameters analyzed at day 21 of pregnancy showed a similar trend to those found at day 20 of gestation. Thus, although glycemia showed no differences between the three groups (Figure 2A), both groups of dams from fructose-fed mothers (FC and FF) had a clear, but non-significant, increase in plasma insulin levels when compared to control dams (CC) (Figure 2B). In fact, when the insulin sensitivity index (ISI) was calculated, both FC and FF groups displayed an evident decrease in this parameter in comparison to the CC group, becoming significant for FF versus control dams (CC) (Figure 2C).

Curiously, although leptin levels were not different between CC and FC dams after fasting and refeeding (Figure 1E,F), leptinemia tended to be higher in FC and it was significantly greater in FF mothers when compared to control ones (CC) at the 21st day of gestation (Figure 2D). Adiponectin, another adipokine, showed the same profile as leptin levels (Figure 2E), although, in this case, the effect was significant for both groups of dams from fructose-fed mothers (FC and FF) versus the CC group. Consequently, Leptin/Adiponectin relation (LAR), a ratio that has been directly linked to situations of insulin resistance and metabolic syndrome disease [37,38], turned out to be higher in both groups of pregnant rats whose mothers had consumed fructose during pregnancy in comparison to CC dams (Figure 2F).

**Figure 3:**
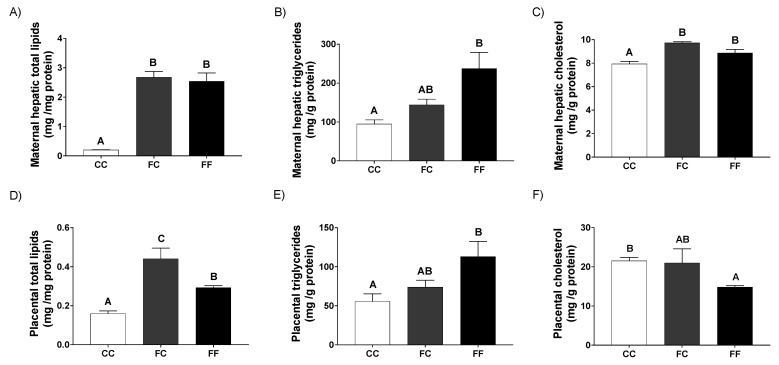
Pregnancy in progeny from fructose-fed mothers and ingestion of a 10% wt/vol fructose solution throughout gestation in pregnant rats from fructose-fed mothers influence maternal liver and placental lipid accumulation. Hepatic content of (**A**) total lipids, (**B**) triglycerides, and (**C**) cholesterol of 21-day-pregnant rats from control mothers (CC, empty bar), or pregnant rats from fructose-fed mothers subjected (FF, black bar) or not (FC, grey bar) to fructose intake throughout pregnancy. Placental content of (**D**) total lipids, (**E**) triglycerides, and (**F**) cholesterol of 21-day-pregnant rats from control mothers (CC, empty bar), or pregnant rats from fructose-fed mothers subjected (FF, black bar) or not (FC, grey bar) to fructose intake throughout pregnancy. Data are mean ± S.E. from 5 pregnant rats. Values not sharing a common letter are significantly different (*p* < 0.05) between the three groups.

Interestingly, both pregnant rats from fructose-fed mothers that consumed fructose (a well-recognized lipogenic substrate) during their pregnancy (FF) and pregnant rats from fructose-fed mothers that did not receive this fructose supplementation (FC), presented a significant and marked liver steatosis (Figure 3A) measured as total lipids in comparison to the control dams (CC). However, whereas this steatosis was due to both an increase in liver triglycerides and cholesterol content in the FF group (Figure 3B), in the FC group it was mainly related to the cholesterol concentration (Figure 3C). These effects are in consonance with the increased hepatic gene expression of transcription factors and enzymes involved in lipogenesis found in FC and, mostly, in FF groups (Table 2). Thus, the mRNA levels of the sterol response element-binding protein-1c (SREBP1c), a typical transcription factor regulating lipogenesis, tended to be increased in both groups of dams from fructose-fed mothers, although without reaching statistical significance. According to this, the expression of lipogenic genes, such as stearoyl-CoA desaturase 1 (SCD1), fatty acid synthase (FAS), and ATP-citrate lyase, tended to be augmented in dams from fructose-fed mothers (Table 2), the effect being more evident in the FF group, and in a clear consonance with the findings observed in hepatic triglycerides (Figure 3B). Moreover, related to this, in FF pregnant rats, but not in FC dams, a clear lower expression of carnitine palmitoyl transferase I (CPT1) was observed when compared to the other two groups, indicating a diminution of hepatic fatty acid catabolism (Table 2). On the other hand, and more related to the findings observed in hepatic cholesterol concentration (Figure 3C), the gene expression of the key enzyme in cholesterol synthesis, 3-hydroxy-3-methyl-glutaryl-CoA (HMG-CoA) reductase, was higher in FC dams, this effect being significant versus the FF group (Table 2).

Similar to the results found in liver, pregnant rats from fructose-fed mothers displayed a clear lipid accumulation in placenta (Figure 3D), this being more evident in FC than FF when measured as total lipids. This accretion of lipids was mainly due to an accumulation of triglycerides since this content tended to be augmented in FC and FF rats, being significantly different in FF versus CC (Figure 3E). On the other hand, the cholesterol content was not changed in FC in comparison to the CC group and, surprisingly, it was reduced in FF (Figure 3F). In relation to this, placental gene expression of lipogenic genes seemed to be more related to the findings observed for total lipids (Figure 3D), since it was augmented in FC in comparison to the other two groups, although the effect was not significant in any case (Table 2). For the gene related to fatty acid oxidation (CPT1), a trend to decrease in dams from fructose-fed rats was found and, in contrast, for the gene related to cholesterogenesis (HMG-CoA reductase), a trend to increase in FC and FF versus CC dams was observed (Table 2).

### 3.2. Pregnancy in Progeny from Fructose-Fed Mothers with or without Fructose Intake throughout Gestation Modifies the Insulin and Leptin Responses, Lipid Contents, and Oxidative Stress of Their Fetuses

Once we demonstrated that both directly (FF) and indirectly (FC) maternal fructose intake can affect insulin and leptin responses and lipid accumulation, we wanted to evaluate if their fetuses would also be affected.

**Figure 4:**
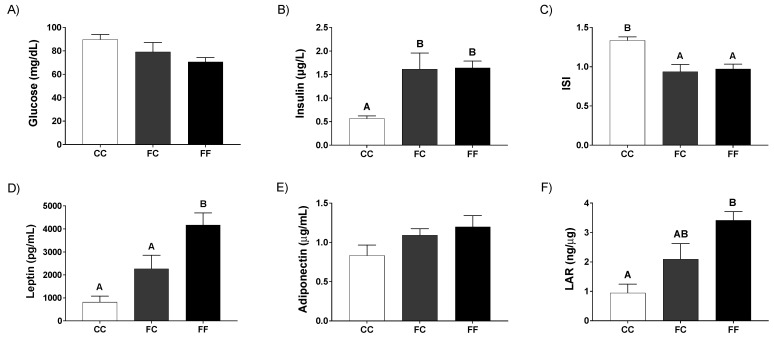
Pregnancy in progeny from fructose-fed mothers and ingestion of a 10% wt/vol fructose solution throughout gestation in pregnant rats from fructose-fed mothers affect fetal leptin and insulin responses. Plasma (**A**) glucose and (**B**) insulin, and (**C**) insulin sensitivity index (ISI) of fetuses from 21-day-pregnant rats from control mothers (CC, empty bar), or pregnant rats from fructose-fed mothers subjected (FF, black bar) or not (FC, grey bar) to fructose intake throughout pregnancy. Plasma (**D**) leptin and (**E**) adiponectin, and (**F**) leptin/adiponectin ratio (LAR) of fetuses from 21-day-pregnant rats from control mothers (CC, empty bar), or pregnant rats from fructose-fed mothers subjected (FF, black bar) or not (FC, grey bar) to fructose intake throughout pregnancy. Data are mean ± S.E. from n = 5 plasma pools of the fetuses of the same litter. Values not sharing a common letter are significantly different (*p* < 0.05) between the three groups.

Thus, although a slight reduction can be observed in glycemia of fetuses from FC and FF mothers, this parameter did not change significantly when compared to control CC (Figure 4A). However, interestingly, insulinemia was significantly increased in fetuses from FC and FF dams (Figure 4B). Accordingly, the insulin sensitivity index turned out to be clearly and significantly reduced in fetuses from pregnant rats from fructose-fed mothers (FC and FF) versus fetuses from control dams (CC) (Figure 4C). Regarding leptinemia, although there was a tendency to rise in fetuses from both FF and FC mothers, this effect was only significant in the FF group (Figure 4D), and this profile was coincident to the one found for their respective mothers (Figure 2D). These results could be due to the fact that this hormone can cross the placenta [39]. Adiponectinemia did not change between the three groups (Figure 4E). Accordingly, the LAR relation showed the same profile to the one observed in leptinemia, that is, a significant increase in fetuses of the FF group, while the increase did not become significant in the FC fetuses (Figure 4F). 

**Figure 5:**
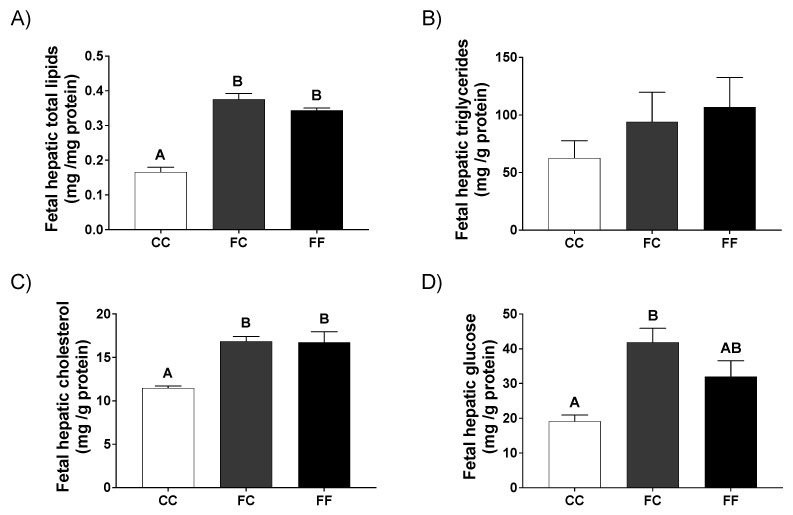
Pregnancy in progeny from fructose-fed mothers and ingestion of a 10% wt/vol fructose solution throughout gestation in pregnant rats from fructose-fed mothers influence fetal liver lipid accumulation. Hepatic content of (**A**) total lipids, (**B**) triglycerides, (**C**) cholesterol, and (**D**) glucose of fetuses from 21-day-pregnant rats from control mothers (CC, empty bar), or pregnant rats from fructose-fed mothers subjected (FF, black bar) or not (FC, grey bar) to fructose intake throughout pregnancy. Data are means ± S.E. from *n* = 5 liver pools of the fetuses of the same litter. Values not sharing a common letter are significantly different (*p* < 0.05) between the three groups.

Surprisingly and in accordance with the findings observed in their respective mothers, livers of fetuses from dams whose mothers had consumed fructose during pregnancy, showed lipid accumulation. Thus, significantly higher levels of hepatic total lipids (Figure 5A) were observed in fetuses from FC and FF dams in comparison to fetuses from CC pregnant rats. Both triglyceride content (Figure 5B) and cholesterol levels (Figure 5C) were increased in fetal liver of FC and FF dams versus pups from CC mothers, although only the cholesterol content reached statistically significant differences. Moreover, fetal liver from FC and FF pregnant dams presented elevated glucose contents which, curiously, reached significant values in the FC group (Figure 5D) versus fetuses from CC rats. These results reinforce the idea that the liver of fetuses from fructose-fed mothers was clearly altered, regardless of the treatment that these dams received during their own gestation (FC and FF).

In this sense, triglyceridemia and plasma NEFA levels showed opposite results to those found for hepatic lipid levels (Figure 5A,B). Thus, plasma triglycerides and free fatty acid levels tended to be reduced, although not significantly, in fetuses from dams of fructose-fed mothers, whereas cholesterolemia did not exhibit any change between the three groups of fetuses evaluated (Table 3). In a previous study, we had already observed that fetuses from fructose-fed mothers (CF) tended to be hypotriglyceridemic [23]. Regarding fetal liver gene expression, whereas mRNA levels of the transcription factor SREBP1c remained unchanged between the three groups, gene expression of the lipogenic enzymes (FAS, ATP citrate lyase and SCD1) tended to be increased in fetuses from dams of fructose-fed mothers, although without being significant in any case. In contrast, the gene expression of the catabolic enzyme CPT1 tended to be reduced (Table 3) in fetuses from FC and FF dams. All these results are in accordance with the liver steatosis mentioned above for these groups (Figure 5A,B). However, despite the higher content of cholesterol found in the liver of fetuses from dams of fructose-fed mothers, HMG-CoA gene expression was not different between the three groups (Table 3).

**Figure 6:**
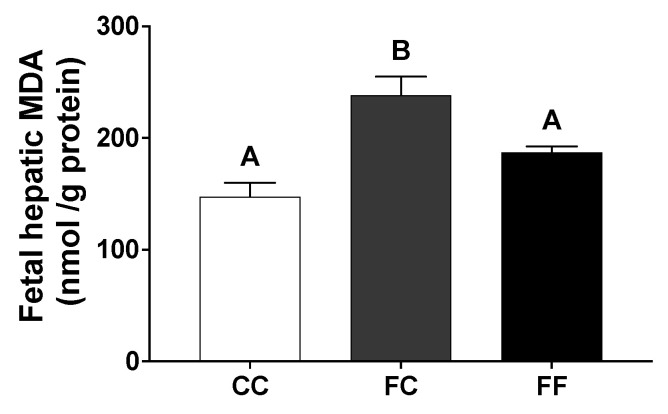
Pregnancy in progeny from fructose-fed mothers produces higher fetal hepatic oxidative stress than ingestion of a 10% wt/vol fructose solution throughout gestation in pregnant rats from fructose-fed mothers. Hepatic MDA values of fetuses from 21-day-pregnant rats from control mothers (CC, empty bar), or pregnant rats from fructose-fed mothers subjected (FF, black bar) or not (FC, grey bar) to fructose intake throughout pregnancy. Data are mean ± S.E. from *n* = 5 liver pools of the fetuses of the same litter. Values not sharing a common letter are significantly different (*p* < 0.05) between the three groups.

Remarkably, an unexpected finding was observed when lipid peroxidation products were measured as MDA levels. A significantly higher hepatic lipid peroxidation was observed in fetuses of the FC group when compared to fetuses of the other two groups (CC and FF) (Figure 6), clear evidence that the livers of fetuses of pregnant rats from fructose-fed mothers (FC) were already unhealthy even without having subjected their mothers to the re-exposure of liquid fructose.

## 4. Discussion

In previous reports, we have observed that whereas fructose intake during pregnancy altered insulinemia and insulin signaling in male descendants, no effects were found in adult female progeny [27]. However, after re-exposure of these females to fructose a marked dyslipidemia and steatosis were observed [28]. It is well known that pregnancy is a physiological status that produces profound changes in lipids and glucose metabolism, and insulin and leptin signaling. Therefore, in the present study, we wanted to establish if pregnancy would be sufficient to provoke metabolic disturbances in these females from fructose-fed mothers as significantly as was observed after fructose re-exposure.

As previously described, fructose intake in drinking water during pregnancy led to an increase in liquid consumption along with a diminution in solid food intake in order to compensate the total calorie intake [23,40]. Curiously, in the present study, although total calories ingested by FF dams during gestation were higher than the calorie intake by CC or FC groups, body weight at day 21 of pregnancy and body weight increase during gestation did not show changes between the three experimental groups. Similar results related to the higher liquid intake and the enhanced total amount of ingested energy due to fructose consumption during gestation with no changes in maternal body weight have been previously recorded in rodents [24,41]. Interestingly, no changes in the amount of chow or liquid diet ingested nor body weight during gestation were seen in FC pregnant rats when compared to controls (CC), which means that maternal fructose intake does not seem to affect the progression of gestation in the progeny. In fact, none of the pregnant descendants from fructose-fed mothers showed changes in conceptus weight, the number of fetuses or fetal body weight, regardless of the treatment received during their own pregnancy (FC and FF). Školníková et al. [42] observed neither modifications in fetal body weight or the number of fetuses in a previous intergenerational study with a 70% sucrose solid diet.

Regarding the glucose tolerance test and insulin sensitivity study, we obtained two interesting findings: (1) Fructose at 10% *w/v*, (concentration that resembles the one found in SSBs and juices) [43,44], caused clear disturbances in the OGTT since a higher production of insulin was required in FF dams in response to the bolus of glucose to regulate glucose levels. Curiously, we had observed in a previous report [23] in descendants from control mothers, that fructose intake during pregnancy (CF) did not produce any differences in glucose tolerance and insulin sensitivity versus the control group (CC). In agreement with the results found in the present study, Song et al. [13] gave 10% fructose in drinking water before and throughout pregnancy and then, during lactation, both to pregnant rats and their descendants when they also got pregnant. They found that fructose feeding caused hyperinsulinemia and insulin resistance to a greater extent in F1 than in F0. Furthermore, (2) interestingly, AUC of plasma insulin concentration during OGTT was higher in the two groups of pregnant rats from fructose-fed mothers (FC and FF) than in controls (CC), which means that it was necessary to produce more insulin to respond to the bolus of glucose but, while FC dams were able to manage glucose levels, FF pregnant rats did not. In accordance with this, insulin levels at day 21 of gestation showed a tendency to increase and, consequently, ISI was diminished both in FC dams and FF when compared to control pregnant rats. Therefore, pregnancy was a sufficiently strong enough change to affect insulin sensitivity in fructose-fed descendants. Moreover, both hyperinsulinemia and the impairment of insulin response observed in FC and FF pregnant rats were also found, and even more marked, in their fetuses. These results mean that maternal fructose intake did not only affect female progeny during their gestation, but also their fetuses. Furthermore, it is important to remark that these effects are seen in female descendants from fructose-fed mothers which had never been exposed to fructose during their entire lives revealing the key role of fetal programming of fructose, whose deleterious effects appeared when these descendant rats became pregnant.

In a previous report [23], we found that fructose intake during pregnancy provoked deficient leptin signaling in dams in response to refeeding. That is why leptin levels were measured in the OGTT experiment of the present study. Thus, the three groups showed similar fasted leptinemia; however, whereas leptin levels in FC dams almost paralleled those of the control group (CC) in response to refeeding, leptin production was clearly more exacerbated in FF pregnant rats. Curiously, maternal fructose intake in dams from control mothers (CF, in our previous report [23]) provoked elevated leptinemia at fasting and no response to refeeding while, in contrast, maternal fructose intake to dams from fructose-fed mothers (FF, present study) produced an exaggerated response to a bolus of glucose. That means that fructose intake during pregnancy must produce some kind of modification in female pups that induces a more pronounced response of leptin levels after refeeding in these rats, when adults. Nevertheless, Levy et al. [45] proposed that both blunted and exaggerated acute leptin secretory responses are inadequate reactions. Thus, no response may result in, for example, obesity, while an exaggerated signal may indicate leptin resistance. Fructose is known to produce hyperleptinemia and leptin resistance [35,36], but no changes in leptinemia were previously reported by our group in non-pregnant female descendants from fructose-fed mothers regardless of whether they were further supplemented with fructose or not [22,28]. Remarkably, at day 21 of pregnancy, dams from fructose-fed mothers had elevated plasma levels of leptin versus CC group. Since fasting leptinemia has been related to body fat stores, the hyperleptinemia observed in FC and FF could not be attributed to a higher adiposity because fasting leptinemia was similar between the three groups. Therefore, it could possibly be more related to the lower insulin sensitivity found in FC and FF versus the CC group. Several examples of dysregulation of the adipoinsular axis have previously been reported by us in descendants from fructose-fed mothers [22,27]. Surprisingly, levels of adiponectin, another adipokine, were increased in pregnant rats from fructose-fed mothers regardless of the treatment that they had received during pregnancy. Although reductions in adiponectinemia have been related to insulin resistance in rats receiving fructose or high-fat diets [40,41], we have previously reported increments in adiponectin levels both in fructose-fed pregnant rats [26] and in non-pregnant descendants from fructose-fed mothers with and without re-exposure to fructose [29,30]. Since it is known that adiponectin is able to improve insulin sensitivity [39], we attributed these increments in adiponectinemia to an attempt to preserve the insulin sensitivity in the livers of these animals. Interestingly, the tendency of leptinemia to be elevated in both FC and FF groups was also observed in their offspring. Given that rat placenta appears to be permeable to leptin, it is logical to find that the levels of this hormone in the fetal circulation reflect those of their respective mothers [39].

Fructose is a well-recognized lipogenic substrate; therefore, it is striking to find a clear accretion of lipids in the liver and placenta of dams from fructose-fed mothers, regardless of whether they received fructose (FF) or not (FC) during pregnancy. This accumulation of lipids, in the case of FC was mostly due to an increase in the cholesterol content whereas in the FF group was clearly due to an accretion of triglycerides. In accordance with that, gene expression of typical lipogenic enzymes and transcription factors (SREBP1c, FAS, ATP citrate lyase, and SCD1) tend to increase in FC and FF dams in a similar way as the triglyceride content, while HMGCoA reductase gene expression seemed to match the findings found in liver cholesterol concentration. Coincident to this, it is known that insulin inhibits gluconeogenesis and activates lipogenesis in the liver [46] but, while in situations of insulin resistance glucose is continuously produced, lipid synthesis is not compromised [47]. In fact, the hepatic glucose content was significantly increased both in FC and FF dams (34.32 ± 1.87, 48.35 ± 2.74, and 48.36 ± 2.05 mg/g of protein for CC, FC and FF groups, respectively; *p* < 0.05 FC and FF versus CC group).

Interestingly, lipid accretion was also seen in the liver of fetuses from fructose-fed mothers, no matter if they had or had not received fructose during their own pregnancy. Again, it was remarkable that this hepatic lipid accumulation was observed both in fetuses from dams receiving fructose (FF) in drinking water and in those pups whose mothers ingested only water (FC). Thus, gene expression of lipogenic and fatty acid oxidation enzymes explained the accumulation of lipids observed in fetal liver of FC and FF dams. These findings could be related to the lower insulin sensitivity found in fetuses of dams from fructose-fed mothers (FC and FF). Nevertheless, leptin has been proposed as being able to control intracellular triglyceride homeostasis by increasing fatty acid oxidation and decreasing fatty acid synthesis and triglyceride accumulation [48,49]. Therefore, in the fetuses from FF dams in which plasma leptin levels were elevated and none of these effects attributed to leptin [23] were observed, a state of leptin resistance could also be involved.

Accumulating evidence suggests that several vitamin imbalances such as inadequate cobalamin status or folate deficiency during pregnancy will affect fetal programming mechanisms, resulting in insulin resistance in both offspring and mothers [50]. Interestingly, several derivatives of inositol have been classified as insulin-sensitizers and seem to counteract insulin resistance-related metabolic diseases with a safe nutraceutical profile. Thus, supplementation with inositol derivatives might act in synergy with other insulin sensitizing drugs and/or nutraceuticals such as lipoic acid and N-acetyl cysteine [51]. Related to that, we previously observed decreased plasma folic acid levels and increased plasma homocysteine in non-pregnant descendants from fructose-fed mothers [52]. However, in the present study, although several characters of MetS were found in pregnant rats from fructose-fed dams, plasma homocysteine levels did not differ between the three experimental groups (8.7 ± 0.3, 7.9 ± 0.3, 8.0 ± 0.6 μM for CC, FC and FF mothers, respectively).

Remarkably, fructose ingested by grandmothers during pregnancy is sufficient to provoke clear harmful effects in the fetuses of their descendants, including a higher lipid accumulation in the fetal liver accompanied with a higher hepatic lipid peroxidation (measured as MDA) in fetuses of FC dams. Coincident to the findings previously found by us in fetuses from fructose-fed mothers (CF) [53] and possibly related to the high reactivity of fructose that their mothers were receiving, a tendency to greater lipid peroxidation was also observed in fetuses of FF dams. Interestingly, the profile observed for the levels of lipid peroxides in fetuses (Figure 6) was clearly coincident with the profile found for hepatic glucose content (Figure 5D). Related to this, Otero et al. [54] have demonstrated that glucose can accelerate the rate of lipid oxidation and this effect is concentration-dependent.

## 5. Conclusions

In conclusion, we can confirm that fructose intake during pregnancy generates a programmed phenotype in the progeny that is initially hidden, but which becomes evident when these descendants are pregnant demonstrating that pregnancy is sufficient to reveal such a programmed phenotype. Indeed, pregnancy produces detrimental effects in these descendants such as alterations of insulin and leptin signaling pathways, and lipid accumulation. Therefore, fructose intake during pregnancy provokes some kind of fetal programming that affects the metabolic health of the progeny when adult and, even more worrying, it can also affect normal pregnancy development in the descendants. In fact, their fetuses (future F2 generation) showed the same deleterious effects found in their mothers. To our knowledge, this is the first time that an intergenerational effect of fructose (10% p/v) intake only in pregnancy has been reported. Interestingly and in accordance with our previous reports, the programmed phenotype induced by maternal fructose intake exists and is revealed either by a re-exposure to fructose in non-pregnant descendants or only with pregnancy. The effects found in mothers which did not receive fructose (FC) were so impressive that fructose ingestion by these pregnant offspring (FF) was only able to produce a slightly higher severity, in terms of leptin function and steatosis. These results confirm the key role of nutrition during pregnancy and the clear relationship between the maternal diet and the development of metabolic diseases in the offspring. In future reports, it will be important to study the metabolic profile of the F2 generation when adults, to confirm that the detrimental effects found in the fetuses remain in adulthood.

## Figures and Tables

**Table 1:** Body weight, food, and liquid ingestion in pregnant rats from fructose-fed mothers subjected (FF) or not (FC) to fructose intake throughout pregnancy and control mothers (CC).

	CC	FC	FF
Maternal body weight at day 0 (g)	203.2 ± 0.2 ^B^	180.0 ± 5.7 ^A^	176.5 ± 1.5 ^A^
Maternal body weight at day 21 (g)	364.0 ± 6.1	339.4 ± 7.1	334.8 ± 8.3
Maternal body weight increase (conceptus free) (g)	161.0 ± 5.0	159.4 ± 5.6	156.1 ± 7.7
Conceptus weight (g)	87.6 ± 8.1	87.2 ± 4.7	79.8 ± 5.8
Number fetus/litter	11.8 ± 1.2	11.3 ± 1.0	10.8 ± 1.0
Fetal body weight (g)	5.6 ± 0.1	5.9 ± 0.4	5.6 ± 0.1
AUC consumed diet (g/21 days per rat)	449.6 ± 16.6	437.1 ± 12.5	371.1 ± 18.5
AUC ingested liquid (mL/21 days per rat)	635.0 ± 41.7 ^A^	699.4 ± 48.2 ^A^	1479.7 ± 139.7 ^B^
Total amount of ingested energy (Kcal/21 days per rat)	1304.0 ± 48.2 ^A^	1267.4 ± 36.3 ^A^	1668.2 ± 28.7 ^B^

Data are expressed as mean ± S.E., *n* = 5 rats. Different letters indicate significant differences between the groups (*p* < 0.05).

**Table 2:** Liver and placental gene expression in pregnant rats from fructose-fed mothers subjected (FF) or not (FC) to fructose intake throughout pregnancy and control mothers (CC).

	CC	FC	FF
	Maternal liver mRNA gene expression (a.u.)		
SREBP1c	0.94 ± 0.20	1.21 ± 0.36	1.52 ± 0.46
FAS	18.9 ± 2.1	25.7 ± 5.8	38.0 ± 11.8
ATP citrate lyase	0.230 ± 0.041	0.463 ± 0.106	0.536 ± 0.175
SCD1	0.79 ± 0.08	1.34 ± 0.41	2.31 ± 0.75
CPT1	0.760 ± 0.025 ^AB^	0.900 ± 0.239 ^B^	0.273 ± 0.063 ^A^
HMG-CoA Reductase	0.59 ± 0.10 ^AB^	1.03 ± 0.32 ^B^	0.32 ± 0.02 ^A^
	Placental mRNA gene expression (a.u.)		
SREBP1c	0.140 ± 0.016	0.175 ± 0.010	0.112 ± 0.020
FAS	0.568 ± 0.010	0.735 ± 0.120	0.526 ± 0.060
ATP citrate lyase	0.056 ± 0.002	0.060 ± 0.004	0.060 ± 0.004
SCD1	0.0015 ± 0.0003	0.0035 ± 0.0010	0.0011 ± 0.0001
CPT1	0.044 ± 0.007	0.033 ± 0.005	0.026 ± 0.003
HMG-CoA Reductase	0.070 ± 0.007	0.095 ± 0.010	0.106 ± 0.014

Data are expressed as mean ± S.E., *n* = 5 rats. Different letters indicate significant differences between the groups (*p* < 0.05).

**Table 3:** Plasma analytes and liver gene expression in fetuses from pregnant rats from fructose-fed mothers subjected (FF) or not (FC) to fructose intake throughout pregnancy and fetuses from control mothers (CC).

	CC	FC	FF
	Fetal plasma levels		
Triglycerides (mg/dL)	16.3 ± 4.4	12.2 ± 1.2	10.2 ± 1.8
Cholesterol (mg/dL)	58.8 ± 2.1	61.5 ± 3.5	60.4 ± 3.5
NEFA (mmol/L)	0.084 ± 0.014	0.064 ± 0.010	0.053 ± 0.005
	Fetal liver mRNA gene expression (a.u.)		
SREBP1c	0.510 ± 0.062	0.545 ± 0.081	0.506 ± 0.043
FAS	4.20 ± 0.87	4.68 ± 1.70	4.85 ± 0.35
ATP citrate lyase	0.158 ± 0.022	0.170 ± 0.037	0.170 ± 0.009
SCD1	0.088 ± 0.008	0.138 ± 0.058	0.143 ± 0.010
CPT1	0.436 ± 0.111	0.333 ± 0.085	0.302 ± 0.024
HMG-CoA Reductase	0.628 ± 0.148	0.663 ± 0.221	0.614 ± 0.035

Data are expressed as mean ± S.E., *n* = 5 plasma pools of the fetuses of the same litter, and RNA was prepared from liver pools of fetuses of the same litter (*n* = 5).

## Data Availability

All data generated or analyzed during this study are available from the corresponding author on reasonable request.

## Abbreviations

The nomenclature used to specify the experimental groups is as follows: The first letter indicates whether the mothers (F0 generation) were supplied with tap water (C, control) or water containing 10% of fructose (F, fructose) during pregnancy; and the second letter indicates whether the progeny (F1 generation) received fructose (F) or not (C) during their own pregnancy.
